# Association between long-term PM_2.5_ exposure and mortality on Sumatra Island: Indonesian Family Life Survey (IFLS) 2000–2014

**DOI:** 10.1007/s10661-024-13323-5

**Published:** 2024-11-06

**Authors:** Sepridawati Siregar, Nora Idiawati, Abiyu Kerebo Berekute, Muchsin Maulana, Wen-Chi Pan, Kuo-Pin Yu

**Affiliations:** 1 Faculty of Medicine, Abdurrab University, Pekanbaru, Indonesia; 2 Institute of Environmental and Occupational Health Sciences, National Yang Ming Chiao Tung University, Taipei, Taiwan; 3 Faculty of Math and Science, Tanjungpura University, Pontianak, Indonesia; 4 Department of Chemistry, College of Natural and Computational Sciences, Arba Minch University, Arba Minch, Ethiopia; 5 Institute of Public Health, National Yang Ming Chiao Tung University, Taipei, Taiwan

**Keywords:** Particulate matter, Natural causes, Cardiovascular causes, Respiratory causes, Mortality

## Abstract

The concentration of PM_2.5_ (particulate matter with a diameter < 2.5 µm) on Sumatra Island has increased, mainly because of forest and peatland fires, transportation, and industry. Biomass burning releases partially burned carbon into the atmosphere, resulting in a smoky haze containing PM_2.5_. Air quality has deteriorated quickly, and PM_2.5_ has become a major health hazard in Indonesia. Studies on long-term exposure to PM_2.5_ have indicated its associations with both morbidity and mortality. Here, we measured long-term (2000–2014) exposure to PM_2.5_ on the basis of satellite-derived aerosol optical depth measurements (1 × 1 km^2^) used to predict ground-level PM_2.5_ concentrations. Additionally, population data on Sumatra Island residents from the fourth wave of the Indonesian Family Life Survey (IFLS) were obtained. We investigated the association between long-term PM_2.5_ exposure and mortality with a retrospective cohort study design. A total of 2409 subjects aged ≥ 40 years participated in the IFLS-3 beginning in November 2000, and we examined mortality outcomes until the IFLS-5 in September 2014. We used Cox regression models to calculate hazard ratios (HRs) of mortality associated with PM_2.5_ exposure. According to the adjusted model, the mortality HRs per 10 µg/m^3^ increase in PM_2.5_ concentration were 1.10 (95% CI 1.03, 1.17) for all natural causes, 1.17 (95% CI 1.05, 1.25) for cardiovascular causes, and 1.19 (95% CI 1.04, 1.36) for respiratory causes. Long-term exposure to PM_2.5_ was associated with all-natural, cardiovascular, and respiratory mortality on Sumatra Island, where PM_2.5_ levels exceed the WHO and US-EPA air quality standards.

**Supplementary Information:**

The online version contains supplementary material available at 10.1007/s10661-024-13323-5.

## Introduction

Exposure to air pollution is associated with increased cardiovascular mortality and morbidity (Cesaroni et al., 2014; Patanè, 2020; Tibuakuu et al., 2018). Globally, particulate air pollution is suggested to be responsible for > 4 million deaths every year (Jiang et al., 2016; Thangavel et al., 2022). Notably, particulate matter with a diameter of < 2.5 µm (PM_2.5_) can be deposited in the upper and lower airways. Because of its small size, it can enter the bloodstream via the alveolar capillaries and can reach more deeply into the lungs and circulation, with the potential to cause serious health problems (Anderson et al., 2012; Schulze et al., 2017; Xing et al., 2016). In addition to genetic and lifestyle risk factors, exposure to air pollution, specifically PM_2.5_, has been implicated in deleterious outcomes and has been linked to cardiovascular disease (CVD) and stroke mortality (Hayes et al., 2020; Lee et al., 2018). PM is often considered the most policy-relevant criterion among the World Health Organization criteria because it is emitted from the burning of fossil fuels in road traffic, shipping, and power generation. It affects almost all body organ systems (Babatola, 2018; Perera, 2017).

The mitigation of air pollution is urgently needed in Indonesia, where air quality has deteriorated quickly due to rapid industrialization and urbanization (Kim Oanh et al., 2006; Santoso et al., 2020; Sofia et al., 2020). On Sumatra Island, aerosol pollutants can linger in the environment for weeks to months at elevated levels, contributing to regional pollution (Crippa et al., 2016; Santoso et al., 2022; Sumaryati et al., 2019). There are no studies on the long-term effects of air pollution on mortality in Indonesia because of a lack of direct measurements (Purnomo et al., 2019; Siregar et al., 2022a, 2022b). However, prior to 2015, PM_2.5_ was not a priority in the Indonesian government’s air pollution management strategy (Purnomo et al., 2019). In the present study, long-term PM_2.5_ was estimated via high-resolution satellite data from the National Aeronautics and Space Administration (NASA) via the aerosol optical depth (AOD) satellite. It is possible to use AOD-PM_2.5_ concentration readings to estimate actual ambient PM_2.5_ levels in areas without ground-based air monitors. Studies have confirmed the relationship between AOD measurements and ground-based PM_2.5_ concentrations (Braggio et al., 2020). In addition, we assessed the long-term effects on mortality in a large elderly cohort (the Indonesian Family Life Survey (IFLS)) on Sumatra Island from 2000 to 2014, as most forest and peatland fires in Indonesia occur on this island.

## Materials and methods

### Study population

We included a total of 2409 subjects who participated in the third wave of the IFLS and were recontacted from June through November 2000 (Supplemental Material, Fig. 1S); we also examined mortality outcomes through record linkage to the death registry until September 2014. The adult participants were aged ≥ 40 years, completed the chronic-type conditions (B3B_CD3) questionnaire, and lived on Sumatra Island. This study excluded participants with incomplete information regarding the variables needed for assessment, those with invalid home address information, and those who moved to another city not included in the IFLS area. The complete IFLS data and guidelines are publicly available on the RAND Corporation website (https://www.rand.org/labor/FLS/IFLS/ifls5.html). RAND, Survey Meter, and Gadjah Mada University, which undertook IFLS-4 and IFLS-5, obtained ethical approval for these waves.

### Cohort follow-up and mortality assessment

The baseline survey determined vital status by responses to follow-up questionnaires and other mailings to study participants. Book 3A consisted of Adult Information Part 1 (Retrospective Information), and Book 3B consisted of Adult Information Part 2 (Current Information). We calculated hazard ratios (HRs) for deaths categorized according to the *International Statistical Classification of Diseases, 10th Revision* (ICD-10; WHO 2010) to define deaths due to all-natural causes (codes A00-R99), CVDs (100–99), and respiratory diseases (J00-47, 80–99). Study participants were excluded from the analyses if they died within 1 year of enrollment or were lost to follow-up by the end of 2014.

### Exposure assessment

The annual PM_2.5_ concentrations for Sumatra Island from 2000 to 2014 were derived via a prediction model that is based on satellite AOD measurements. This study employed Quantum Geographic Information System (QGIS) software (version 3.4.15) to process and analyze these AOD measurements. QGIS was used to convert the unitless AOD values into PM_2.5_ concentrations (µg/m^3^) and apply the inverse distance weighted (IDW) interpolation method to visualize the data across Sumatra Island. This open-source GIS software has proven essential for addressing spatial analysis challenges and generating insights into PM_2.5_ distributions (Talib et al., 2022). However, there are certain limitations in predicting PM_2.5_ from AOD. Research has consistently highlighted that smoke from forest fires can lead to overestimation or misinterpretation of PM_2.5_ concentrations derived from AOD. This occurs because the complex interaction between smoke and satellite observations complicates the accurate retrieval of aerosol properties (Braggio et al., 2020; Zhang et al., 2023).

To derive AOD values for the 1-km grid cells, we used the GADM version 2.8 map layer of Indonesia’s administrative areas. The GADM (Global Administrative Areas Database) provides high-resolution data for state administrative regions worldwide, covering all levels and periods (https://gadm.org/). We determined the AOD value for each 1-km grid cell by calculating the area-weighted average of the AOD values from the intersecting administrative polygons. Patients’ geocoded addresses were then matched to the corresponding PM_2.5_ concentration areas on the basis of spatial location and year (Siregar et al., 2022a, 2022b).

Study participants provided detailed address history upon study enrollment and addresses for the year prior; PM_2.5_ predictions were averaged for the year prior for each patient. The data on the exposure of PM_2.5_ concentrations from the QGIS software are provided in the Supplemental Material, Table S1.

### Statistical analysis

The association of PM_2.5_ (2000–2014) with mortality was examined via proportional hazards regression models adjusted for a wide range of individual risk factors. Plots of cumulative log hazard against log survival time for categories of the explanatory variables indicated that the proportional hazards assumption held well. Both total and cause-specific mortality were assessed. Our basic models included age only. In the adjusted models, we added factors that we identified a priori as potential confounders, including sex (man, woman), body mass index (BMI), education (low if they had < 12 years of school attainment, high if they had ≥ 12 years of school attainment), smoking status (never, i.e., they never had a smoking habit; current, i.e., they currently had a smoking habit; or former, i.e., they formerly had a smoking habit), socioeconomic (SES) status (poor, average, rich), diabetes (yes/no), and hypertension (yes/no). BMI was treated as a quintile, given its potential nonlinear relationship with mortality. Since diabetes and hypertension might mediate the relationship between particle exposure and mortality, we examined models with and without adjustment for diabetes and hypertension.

We also conducted stratified analyses by age (< 65 and ≥ 65 years) and sex (male and female) to examine the associations between PM_2.5_ and mortality. We categorized BMI into two categories according to the standard definition: normal/underweight (BMI < 25) and overweight/obese (BMI ≥ 25).

A *p* value < 0.05 indicated statistical significance, and R Studio was used to perform all the statistical analyses.

## Results and discussion

### PM_2.5_ concentration retrieved from satellite AOD measurements

**Fig. 1:**
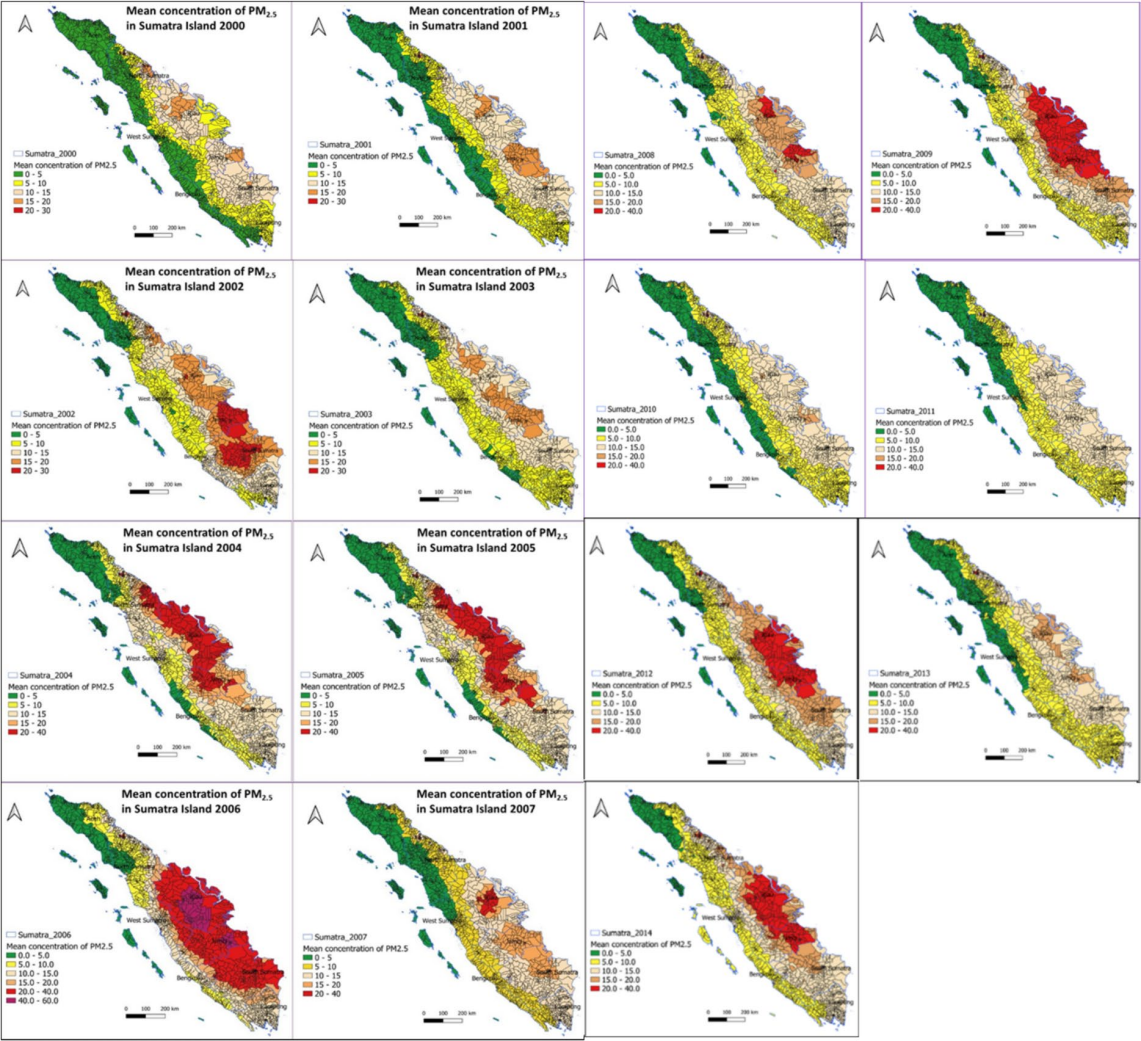
Annual average PM_2.5_ concentrations (2000–2014) on Sumatra Island

**Fig. 2:**
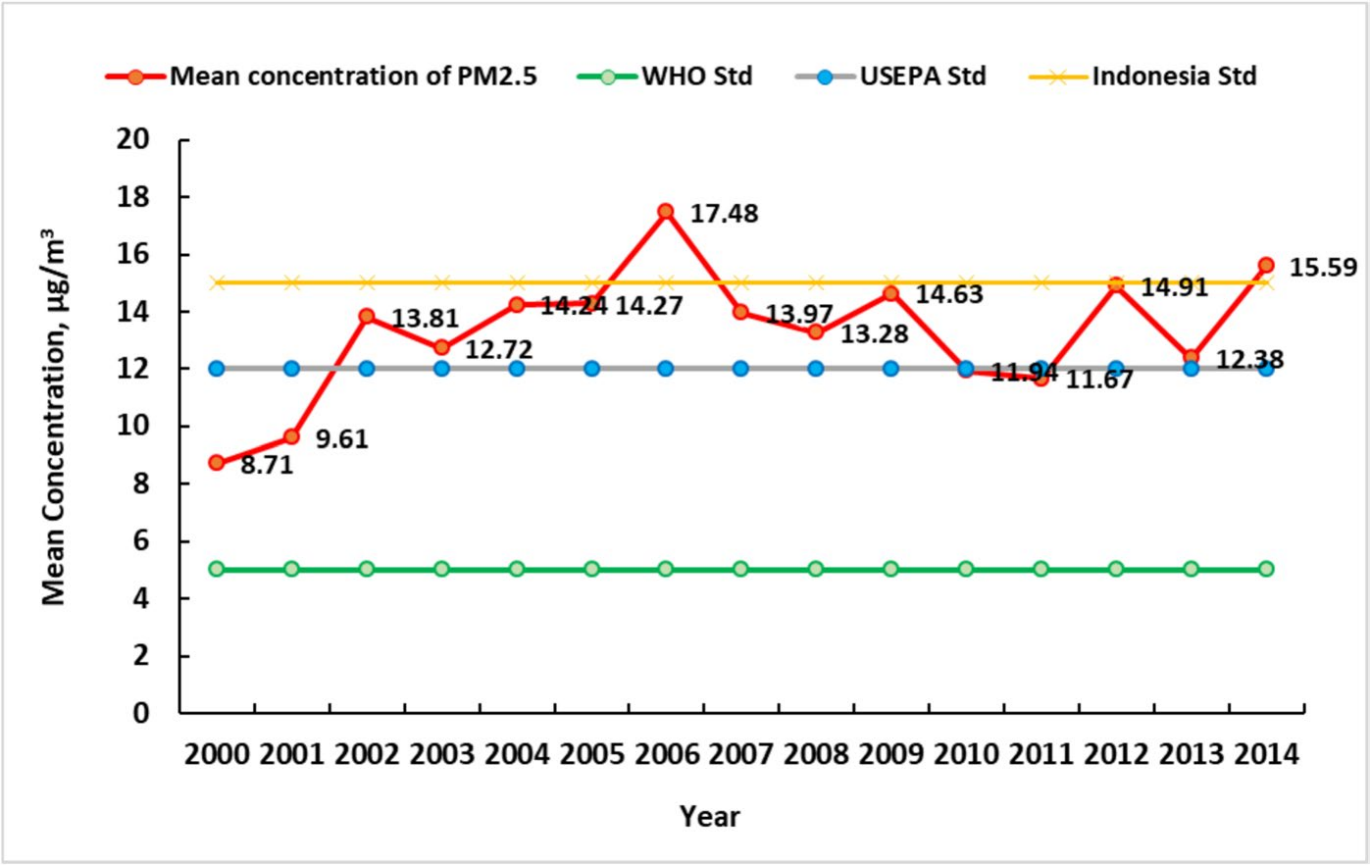
Mean annual PM_2.5_ concentration trend on Sumatra Island from 2000 to 2014 compared with the WHO, US-EPA and Indonesian NAAQS standards

Figure 1 shows the distribution of the mean annual PM_2.5_ concentration from 2000 to 2014 for each 1 × 1 km^2^ grid throughout Sumatra Island, Indonesia. On Sumatra Island, the mean annual PM_2.5_ concentration was lowest in 2000, at 8.71 µg/m^3^, and was highest in 2006, at 17.48 µg/m^3^ (Fig. 2).

On Sumatra Island, the average PM_2.5_ concentration from 2000 to 2014 was 13.3 µg/m^3^. The annual PM_2.5_ concentration generally increased by 40% during the study period from 2000 to 2014, but it increased and then decreased in some years.

### Baseline characteristics

In the first year, 15 deaths were excluded, and 20 participants lost to follow-up by the end of 2014 were excluded. A final sample of 2374 participants was included for PM_2.5_ estimation and analysis. Table 1 presents selected baseline participant characteristics according to whether the subjects died during follow-up.

**Table 1:** Baseline characteristics of 2374 adult participants

Characteristic	Total (*n* = 2374)	Death % (*n* = 487)	Nondeath % (*n* = 1887)
Sex			
Female	1226 (51.6)	49.3	52.8
Male	1148 (48.4)	50.7	47.2
Age			
40–65	1743 (73.4)	35.8	80.5
≥ 65	631 (26.6)	64.2	19.5
BMI			
Under/normal weight (BMI < 25)	1406 (59.2)	56.3	60.6
Overweight/Obese (BMI ≥ 25)	968 (40.8)	43.7	39.4
Education			
Low (if they had < 12 y)	2046 (86.2)	66.1	93.5
High (if they had ≥ 12 y)	328 (13.8)	33.9	6.5
SES			
Low	791 (33.3)	30.3	34.0
Medium	1101 (46.4)	46.5	47.2
High	482 (20.3)	23.2	18.8
Smoking behavior			
Never	1243 (52.4)	30.6	60.7
Current	993 (41.8)	54.2	37.4
Former	138 (5.8)	15.2	1.9
Residency area			
Urban	988 (41.6)	38.4	42.0
Rural	1386 (58.4)	61.6	58.0
Chronic conditions			
Hypertension			
Yes	521 (21.9)	18.3	22.0
No	1853 (78.1)	81.7	78.0
Diabetes			
Yes	97 (2.7)	3.7	2.4
No	2277 (97.3)	96.3	97.6

A total of 487 subjects died during follow-up, among whom 460 died from all-natural causes, 144 from CVDs, 197 from respiratory diseases, 43 from lung cancer, and 27 from external causes (Table 2). As expected, the deceased subjects were male, older, and overweight/obese; were more likely to be current/past smokers; had a higher prevalence of diabetes and hypertension; had lower education levels; had low/medium SES; and lived in rural areas.

**Table 2:** Mortality outcomes after 14 years of follow-up at the end of the study in 2014

ICD-10 codes	Mortality cause	No. of deaths	Percent
A00-R99	All-natural causes	460	94.5
100–99	Cardiovascular diseases	144	29.6
J00–47, 80–99	Respiratory diseases	197	40.5
C34.0–9	Lung cancer	43	8.8
S00-T99	External causes	27	5.5
All included codes	All causes	487	

### *Associations between PM*_*2.5*_* and mortality*

According to both the basic and adjusted models, particulate air pollution was found to be associated with an increased risk of cardiopulmonary mortality (Table 3). In the adjusted model, an increase of 10 µg/m^3^ in PM_2.5_ corresponded to 10% (95% CI 1.03, 1.17), 17% (95% CI 1.05, 1.25), and 19% (95% CI 1.04, 1.36) increased risks of natural, cardiovascular, and respiratory mortality, respectively.

**Table 3:** Hazard ratios (95% CI) of mortality associated with a 10-µg/m^3^ increase in the average PM_2.5_ level at baseline (deaths within the first year were excluded)

Cause of death	Basic model^a^	Adjusted model^b^	Adjusted model^c^
All natural causes	1.16 (1.10, 1.23)**	1.13 (1.05, 1.30)**	1.10 (1.03, 1.17)**
Cardiovascular Diseases	1.23 (1.08, 1.40)**	1.20 (1.07, 1.33)**	1.17 (1.05, 1.25)**
Respiratory Diseases	1.26 (1.22, 1.30)**	1.23 (1.06, 1.42)**	1.19 (1.04, 1.36)*
Lung cancer	1.05 (0.95, 1.16)	1.03 (0.90, 1.18)	1.02 (0.79, 1.32)
External causes	1.04 (0.89, 1.22)	1.02 (0.86, 1.21)	%1.%2 (0.75, 1.36)

^*^p < 0.05; **p < 0.01

^a^Covariates included age

^b^Covariates included age, sex, BMI, education, SES, smoking status, and residence area

^c^Covariates included age, sex, BMI, education, SES, smoking status, residence area, diabetes, and hypertension

In stratified analyses (Table 4), the association between a 10 µg/m^3^ increase in PM_2.5_ and mortality was closer to null in both age groups (< 65 and ≥ 65 years) for all mortality outcomes, with significant differences by age for cardiovascular mortality (HR = 1.33; 95% CI 1.12, 1.58 vs. HR = 1.17; 95% CI 1.00, 1.37; interaction *p* value 0.048) and respiratory mortality (HR = 1.40; 95% CI 1.01, 1.94 vs. HR = 1.11; 95% CI 0.99, 1.24; interaction *p* value 0.035). There is a lack of studies proving consistent differences in associations between PM_2.5_ and any of the outcomes according to sex (Table 4) or education level (see Supplemental Material, Table S2) (Wong et al., 2015).

**Table 4:** Hazard ratio (95% CI) per 10 µg/m^3^ increase in PM_2.5_ in stratified analysis by age or sex with exposure at baseline

Cause of death	Age < 65 years	Age ≥ 65 years	Interaction^a^	Male	Female	Interaction^a^
All natural causes	1.21 (1.05, 1.39)**	1.12 (1.02, 1.23)*	0.08	1.26 (1.06, 1.50)**	1.10 (1.02, 1.19)*	0.84
Cardiovascular	1.33 (1.12, 1.58)**	1.17 (1.00, 1.37)	0.048	1.15 (1.03, 1.29)*	1.29 (1.05, 1.58)*	0.45
Respiratory	1.40 (1.01, 1.94)*	1.11 (0.99, 1.24)	0.035	1.26 (1.02, 1.56)*	1.04 (0.84, 1.29)	0.21
Lung cancer	1.03 (0.92, 1.15)	0.98 (0.91, 1.05)	0.21	1.20 (0.88, 1.64)	1.46 (0.96, 2.22)	0.57
External causes	0.88 (0.48, 1.61)	1.08 (0.86, 1.36)	0.73	1.01 (0.66, 1.55)	1.05 (0.36, 3.06)	0.91

^a^*p* value for the interaction term in the model for the combined dataset

^*^p < 0.05; **p < 0.01

In this study, we showed that greater exposure to ambient PM_2.5_ was associated with mortality from natural (10% increase per 10 µg/m^3^ PM_2.5_) and cardiovascular causes (17% increase) and with mortality due to respiratory disease (19% increase), specifically in people ≥ 65 years of age. This result was slightly greater than that of a meta-analysis of 84 cohort studies that reported a 10% increase (95% CI 1.02, 1.19) in CVD mortality and a 7% increase (95% CI 1.04, 1.09) in all-cause mortality per 10 µg/m^3^ increase in PM_2.5_ (Pranata et al., 2020).

**Fig. 3:**
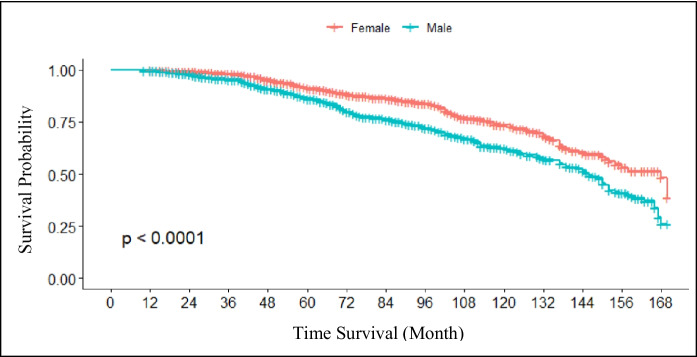
Probability of survival (Kaplan‒Meier) of all causes of death according to sex. The *y*-axis indicates the survival probability along the *x*-axis from baseline to years of follow-up. The differences between sexes were tested by the log-rank test (*p* value < 0.0001)

The survival probability at 168 months is shown in Fig. 3. The results revealed a greater decrease in the survival probability for all causes of death in male participants than in female participants.

The risk we identified for respiratory mortality was similar to that reported in other Chinese studies and higher than that reported in a study in Hong Kong (Li et al., 2019, 2021; Mokoena et al., 2019). The heterogeneity in effect sizes for respiratory mortality among studies may be due to the various characteristics of the study context, such as exposure assessment methodology, air pollution levels and components, and population sensitivity to air pollution. On Sumatra Island, the air pollution sources may differ from those in other countries, with higher air pollution contributions from biomass burning (forest and peatland fires) and motor vehicle exhaust.

In a previous study, we reported that the relative reduction in respiratory mortality was greater in the < 64-year age group than in the ≥ 65-year age group (Hoek et al., 2013; Mokoena et al., 2019; Wang et al., 2017), suggesting heterogeneity of air pollution effects among age groups. In our stratified analysis, participants ≥ 65 years of age were potentially at lower risk from air pollution than those < 65 years, probably due to a healthy survival effect. Particularly for older people, caution is needed when interpreting mortality effects across long study periods, which may vary because of changes in the susceptibility of survivors in different periods of follow-up. Our results revealed no sex differences. Indeed, the current evidence for sex differences in susceptibility is weak and inconsistent among studies (Wong et al., 2015).

Among males and females, respiratory mortality was greater in males than in females. In Indonesia, smoking rates in females are lower than those in males and internationally. Anecdotal evidence suggests that their disinclination to smoke is commonly attributed to cultural values, which stigmatize women smokers as morally flawed while at the same time sanctioning smoking by men (Ayuningtyas et al., 2021; Barraclough, 1999; Holipah et al., 2020).

Compared with association studies of short-term effects, even fewer studies in Indonesia have examined the association between long-term exposure to air pollution and human health. To date, there has been only one published air pollution cohort study in Indonesia (Haryanto & Djafri, 2020).

The results of our study from a satellite-based measure of PM_2.5_ provide new evidence on mortality from the long-term effects of PM_2.5_ exposure. On Sumatra Island, pollution levels are generally heterogeneous; additionally, while our approach applies to an older population with limited mobility, it may be less so for highly mobile populations.

In recent years, Indonesia has been undergoing a stage of transition from economic development to include environmental issues, for which tighter air quality standards are needed. Reliable estimates of the health effects of air pollution from epidemiologic studies are urgently needed to provide essential scientific evidence for environmental accountability and health impact assessments of new air quality objectives. Quantitative knowledge about the relationship between exposure to air pollution and health outcomes is crucial for assessing the health impact of air pollution and its implications for relevant policy-making. Our study fills a critical gap in missing long-term effect estimates for Indonesia and the impact on life expectancy and value of life years, which could be gained because of a reduction in pollutants due to government intervention. The estimates from this study can also support the cost–benefit ratio for achieving clean air and form the basis for important public health information, including air pollution risk communication.

### Strengths and limitations

The main strength of our study was the assessment of all the causes of death according to the ICD-10 codes, which eliminated the possibility of misclassification of cases and measurement errors; however, when interpreting the results, such possible errors should be considered. There are several limitations of our study. First, the pollution levels used were based on city-wide averages (reflecting background exposure levels) rather than personal exposure measurements. Consequently, this was expected to result in exposure measurement error and bias in terms of the precision and strength of the risk estimates. Second, the cause of death was self-reported by the family member and the village head office administration on the basis of the doctor’s diagnosis, and a questionnaire was used to collect information on the covariates. Thus, recall bias was possible, considering that people with a low SES might have been less informed about the exact mortality diagnosis. Third, we could not explore the associations of air pollutants with the causes of mortality subtypes due to the limited detail and number of cases, which involved different pathogens. Fourth, this study did not include alcohol and physical activity because controlling for too many potential confounders can lead to or aggravate data sparsity or multicollinearity problems, particularly when the number of covariates is large in relation to the study size (Greenland et al., 2016). However, from the observation of IFLS data comparing smokers and nonsmokers, the percentage of alcohol and tobacco expenditures is greater in smokers (Dartanto et al., 2018; Martini et al., 2022).

### Policy implications

Our study provides accurate data on CVD and mortality as a result of long-term PM_2.5_ exposure, which is exacerbated by forest and peatland fires, particularly on Sumatra Island, Indonesia. We expect our results to fill the gap in the literature regarding the relationship between long-term exposure to PM_2.5_ and CVD and mortality among adults on Sumatra Island, Indonesia. Our findings may have implications for environmental and social policies and for steps that the Indonesian government can take to protect human health. This study can help raise awareness among health professionals and stakeholders regarding the impacts of PM_2.5_ exposure and may promote the development of policies to address this problem and prevent adverse health outcomes later in life. Owing to deteriorating air quality, these findings also provide advice to the government to maintain long-term local PM_2.5_ data management for all regions in Indonesia so that the data used to support air quality management policies are not as scarce as they are currently available.

Input related to PM_2.5_ quality control efforts can be provided to the Indonesian government in the form of appropriate measures to improve public and environmental health. For example, the Indonesian government needs to develop several technical guidelines under the mandate of **government regulation PP No. 22 of 2021** concerning air pollution control because the national ambient air quality standard policy is still below international standards. In Indonesia, the maximum standard PM_2.5_ in the atmosphere for the mean annual concentration was set at 15 µg/m^3^ and 55 µg/m^3^ for 24 h. According to the USEPA, it is 12 µg/m^3^ for the mean annual concentration and 35 µg/m^3^ for 24 h, and according to the WHO, it is 5 µg/m^3^ for the mean annual concentration. These data can urge the government to immediately implement measures and concrete actions to save forests from extinction and prevent forest and peatland fires.

Hypertension and smoking are the most notable risk factors for stroke and coronary artery disease (Hajar, 2017). The nationwide approach to hypertension prevention and control has contributed to a substantial decline in stroke mortality in Japan (Ohira & Iso, 2013). Recent antismoking campaigns have contributed to a decline in the smoking rate among men (Bala et al., 2017; Chiang & Chang, 2016; Durkin et al., 2009). Intensive prevention programs for hypertension and smoking should continue to prevent future cases of CVD in Indonesia.

### Urgent mitigation recommendation

During forest fire events, safeguarding public health, especially in remote areas, is crucial, and this work is a follow-up study of our previous research (Sepridawati Siregar et al., 2022a, 2022b; Siregar et al., 2022a, 2022b). To address this, several key actions should be taken. First, isolation shelters should be constructed with air filtration systems to remove PM_2.5_ and other pollutants. These buildings need to be well-sealed to prevent smoke infiltration. Shelters should be positioned in easily accessible locations for emergencies and setting up community centers with air filtration for those who do not have personal shelters should be considered. These facilities are equipped with medical supplies, clean drinking water, and communication tools (Xiang et al., 2021). The second, temporary shelters, use portable air purifiers, and air conditioning units to maintain breathable air quality. The filters should be regularly checked and replaced to ensure that they remain effective. The community should be educated about the locations and use of these temporary shelters during fire events (Prathibha et al., 2024). The third goal is to develop early warning systems that integrate satellite data, weather forecasts, and fire detection technologies to provide timely alerts on fire risk and air quality. Localized alert systems can be implemented via mobile apps, text messages, or local radio to inform residents of imminent fire risks and offer instructions on protective measures (Aryanti et al., 2021). Fourth, in terms of health precautions and personal protection, N95 masks should be distributed to reduce PM_2.5_ inhalation and provide guidance on their proper use (Kunstler et al., 2022). The wearing of long-sleeved shirts, hats, and other protective clothing to minimize exposure to smoke and ash should be encouraged. Medical care for individuals with respiratory conditions should be provided, and mobile health units or telemedicine services for remote areas should be set up. Additionally, access to clean water and nutritious food to support overall health and resilience during fire events should be ensured (Kodros et al., 2021).

## Conclusion

In an observation window from 2000 to 2014 and according to an IFLS population-based cohort of participants aged ≥ 40 years, exposure to PM_2.5_ estimated from NASA satellite data in the area of residence was associated with mortality from all-natural, cardiovascular, and respiratory causes. The effect estimates corroborate the existing evidence for a causal relationship between PM_2.5_ and adverse health outcomes and support the formulation and implementation of policies to mitigate PM_2.5_ pollutants and their disease burden.

## Supplementary Information

Below is the link to the electronic supplementary material.

Supplementary file1 (DOCX 86 KB)

## Data Availability

No datasets were generated or analysed during the current study.
