# Supplementary material for: Association between long-term PM2.5 exposure and mortality on Sumatra Island: Indonesian Family Life Survey (IFLS) 2000–2014
Source: Environ Monit Assess. 2024 Nov 6;196(12):1173. doi: 10.1007/s10661-024-13323-5 (PMC11541269; doi:10.1007/s10661-024-13323-5)
Supplement: Supplementary file 1 — Supplementary file1 (DOCX 86 KB) [file 10661_2024_13323_MOESM1_ESM.docx]

**Supplementary Materials**

**Figure S1.** The model impact, molecular and pathophysiological mechanisms of PM_2.5_ on cardiovascular mortality (Feng et al. 2023).


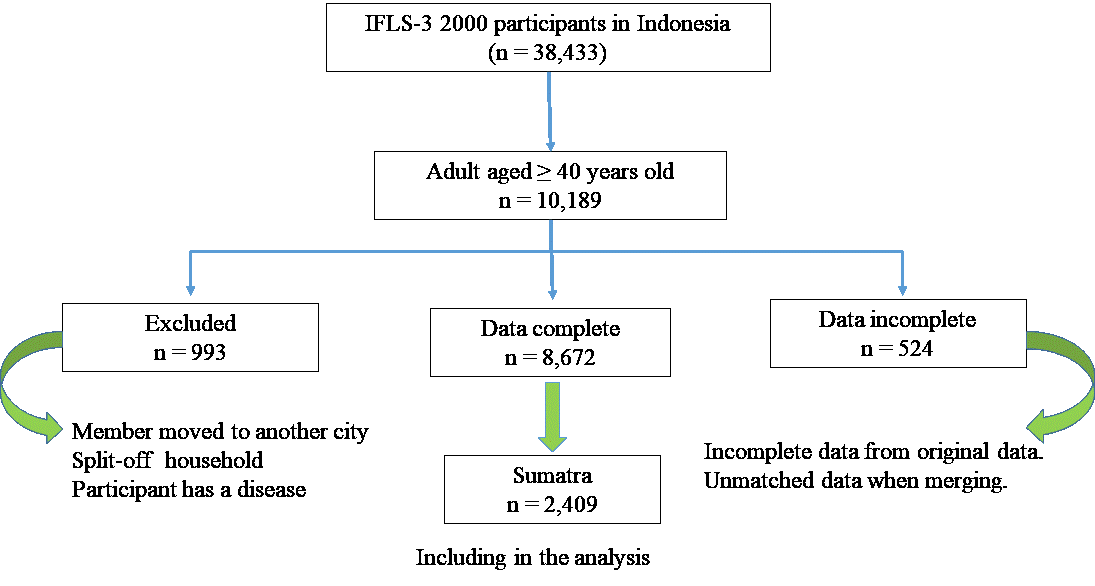


**Figure S2**. The selection study population in Sumatra Island, IFLS-3.

**Table S1.** PM_2.5_ data from QGIS software, in Sumatra Island, 2000-2014.

| Years | Mean annual PM_2.5_ (µg/m^3^) | Maximum PM_2.5_ (µg/m^3^) |
| --- | --- | --- |
| 2000 | 8.71 | 32 |
| 2001 | 9.61 | 31 |
| 2002 | 13.81 | 33 |
| 2003 | 12.72 | 32 |
| 2004 | 14.24 | 44 |
| 2005 | 14.27 | 44 |
| 2006 | 17.48 | 60 |
| 2007 | 13.97 | 32 |
| 2008 | 13.28 | 32 |
| 2009 | 14.63 | 40 |
| 2010 | 11.94 | 31 |
| 2011 | 11.67 | 31 |
| 2012 | 14.91 | 40 |
| 2013 | 12.38 | 32 |
| 2014 | 15.59 | 40 |

**Table S2.** Hazard ratios of mortality associated with 10 µg/m^3^ increase in average PM_2.5_ – stratified by education level.

| Cause of death | Low (<12 y) | High (≥12 y) | Interaction  p-value |
| --- | --- | --- | --- |
| All natural causes | 1.26 (1.06, 1.45)** | 1.12 (1.03, 1.22)* | 0.63 |
| Cardiovascular | 1.32 (1.08, 1.61)** | 1.07 (1.02, 1.12)** | 0.47 |
| Respiratory | 1.21 (1.02, 1.44)* | 1.09 (1.01, 1.18)* | 1.02 |

**p*<0.05; ***p*<0.01.
